# Systemic Sclerosis in Zimbabwe: Autoantibody Biomarkers, Clinical, and Laboratory Correlates

**DOI:** 10.3389/fimmu.2021.679531

**Published:** 2021-11-09

**Authors:** Elopy N. Sibanda, Yvonne Dube, Mazvita Chakawa, Takafira Mduluza, Francisca Mutapi

**Affiliations:** ^1^Department of Pathology, Faculty of Medicine, National University of Science and Technology, Bulawayo, Zimbabwe; ^2^Asthma Allergy and Immunology Clinic, Harare, Zimbabwe; ^3^Department of Pathophysiology and Allergy Research, Medical University of Vienna, Vienna, Austria; ^4^Laboratory Section, Asthma Allergy and Immunology Clinic, Harare, Zimbabwe; ^5^Department of Biochemistry, University of Zimbabwe, Harare, Zimbabwe; ^6^Institute of Immunology and Infection Research, School of Biological Sciences, University of Edinburgh, Edinburgh, United Kingdom

**Keywords:** systemic sclerosis, autoantibodies, clinical, laboratory, respiratory, cutaneous, Zimbabwe

## Abstract

**Introduction:**

Systemic sclerosis (SScl) is an autoimmune disease whose prevalence is rarely reported in Africa. Autoantibodies are the biomarkers of the condition, precede overt disease and determine disease phenotypes. SSc specific autoantibodies also vary between racial groupings. Objective: To investigate the clinical and laboratory characteristics of Zimbabwean patients who were reactive SSc specific autoantibodies.

**Materials and Method:**

240 patients, 173 of them female with SSc specific autoantibodies were included. Autoantibodies were detected by indirect immunofluorescence microscopy and immunoblotting using a panel of 13 SScl (Euroimmun Ag., Germany). Demographic, clinical and laboratory parameters relevant to the monitoring of SScl were captured. These included pulmonary function tests, hematology, clinical chemistry, serology and thyroid function tests. Allergy skin prick tests (SPT) to inhalant and food allergen sources were conducted when indicated.

**Results:**

All the 240 patients (median age was 36 years) expressed SSc specific autoantibodies. 86% were Black, 11% White and 3% Asian and a fifth (20%) were younger than 16 years. Eleven (4.6%) fulfilled the ACR/EULAR classification of SSc. Clinically they had limited cutaneous (n=6), diffuse cutaneous (n=3) and SScl/inflammatory myopathy overlap (n=2). The most frequently detected antibodies anti-RNA polymerase III (RNAP) 55%, anti-Th/To (28%) anti-RNAP 11 (22%), anti-CENPB (18%) and anti-Scl-70/ATA (13%). Racial variations in the expression of these antibodies were apparent between Black, White and Asian patients. The majority (95%), who did not fulfil the ARA/EULAR criteria were symptomatic. Raynaud’s Phenomenon was documented in 24%. Respiratory symptoms included coughing, dyspnea and wheezing. There was a restrictive ventilatory defect with increased FEV1/FVC ratio. Pruritus, urticaria and skin depigmentation were the main cutaneous features while constipation, bloating, Gastroesophageal reflux disease (GERD) and abdominal pain dominated GI symptoms. Mean blood pressure readings while normal varied with biomarkers. Haematology and biochemistry parameters were within normal reference ranges.

**Conclusion:**

The expression of SSc specific autoantibodies is common and associated with known SSc symptoms. The types and frequency of autoantibodies varied with racial groupings. A fifth of the patients were children below the age of 16 years.

## Introduction

Systemic sclerosis (SSc) is a multisystem autoimmune condition that characterized by cutaneous changes, microvascular abnormalities, visceral fibrosis and internal organ dysfunction (1). SSc specific autoantibodies are detected in over 95% of SSc patients and some are included in the international classification criteria for the condition. The diffuse cutaneous (dcSSc) or limited cutaneous (lcSSc) variants are described on the basis of the extent of skin involvement (2). The variants are respectively associated with the SSc specific autoantibodies, anti-topoisomerase I antibodies (ATA) (also known as anti-Scl-70) and anti-centromere antibodies (ACA) whose detection is mutually exclusive. Some patients with anti-RNA polymerase (RNAP) antibodies have the dcSSc phenotype. The skin changes that are universal in advanced disease may be subtle in early disease. Autoantibodies are detectable at all stages and may temporally correlate with the presence of Raynaud’s Phenomenon (RP) a recognized early feature and precursor of SSc. Autoantibodies are diagnostic and predict organ involvement, disease phenotypes and clinical outcomes (3–6). A limited number of autoantibodies are included in the ARA/EULAR classification criteria (7). This SSc classification describes cutaneous changes in symptomatic patients with detectable (anti-ATA, anti-ACA, anti-RNA polymerase III) but does not adequately address non-cutaneous respiratory, gastrointestinal or endocrine symptoms that are early disease features (8).

### Pathology

The pathogenesis of SSc involves interplay between an exposure to unidentified trigger, the processing of such an antigenic trigger by genetically predisposed individuals and an immune response that triggers autoreactivity. The systemic immune response culminates in multi-system endothelial cell proliferation, smooth muscle hypertrophy, myofibroblast activation and extracellular matrix synthesis. Host tissue specific autoantibodies that can precede ARA/EULAR classifiable disease by many years are produced in this immune response cascade (4).

### Clinical Presentation

Early SSc manifestations are non-specific, and depending on affected organs the symptoms can be variously confused with asthma, dermatitis, rheumatic disease or food allergy. Raynaud’s Phenomenon (RP) is one of the early features and, in the presence of SSc-specific autoantibodies predicts the development of SSc in patients (9). The types of autoantibodies detected influences the disease phenotypes and the type and extent of organ involvement. Clinical phenotypes vary with the types of autoantibodies. Some autoantibodies have been associated with malignant conditions.

SSc specific autoantibodies differ with the genetic backgrounds, and (10) are likely to be different in Africans (11). Black ethnicity is associated with a greater frequency of SSc, (12–15) a younger age of onset and more severe disease (16). There are no comparable regional data addressing laboratory and clinical features of patients with detectable SSc specific antibodies. The objective of this study was to profile the clinical and laboratory characteristics of patients with detectable systemic sclerosis specific antibodies.

## Patients and Methods

### Study Design

This was a single center observational study. A review of clinical and laboratory records of patients who had attended the Asthma, Allergy and Immune Dysfunction Clinic, the only clinical immunology referral clinic in Zimbabwe was undertaken.

### Study Population

We included 240 patients that had been referred to the Asthma, Allergy and Immune Dysfunction Clinic in Harare, Zimbabwe between 2013 and 2018 (**Table 1**). The commonest reasons for referral was the evaluation of respiratory, gastrointestinal, cutaneous or other symptoms. Patients with detectable SSc specific and associated serum autoantibodies were included. HIV antibody positive patients were excluded since some can aberrantly produce non-specific autoantibodies.

**Table 1:** Demographic profile of patients.

	Combined data	Under 16 yrs.	Over 16 yrs.	Female: Male (F:M) ratio
All patients (n=240)	240	49 (20%)	191 (80%)	
Age range (years)	<1 - 94	<1 - 16	17 - 94	
Median age (years)	36	7.5 (2.73-12.38)	41.9 (39.8-44.0)	
				
Proportion females	173 (72.1%)	25 (46.5%)	152 (77.6%)	2.4:1
Proportion males	67 (27.9%)	23 (53.5%)	44 (22.4%)	
				
Black patients (n=203)	203	46 (20.7%)	161 (79.3%)	3.5:1
Age range	0-84	<1-16 years	17-84	
Median age (years)	35	7.56 (2.73-12.38)	40	
				
White patients (n=27)	27	1 (3.7%)	26 (96.3%)	3:1
Age range (years)	15-94	15	17-94	
Median age (years)	46	15	47	
				
Asian patients (n=10)	10	2 (20%)	8 (80%)	9:1
Age range (years)	1-60	1-4	22-60	
Median age (years)	31.5	2.5	33.5	

Age, race, and gender distribution of patients with detectable systemic sclerosis-specific autoantibodies.

### Clinical and Laboratory Investigations

Demographic and routine clinical records, temperature and blood pressure and body mass index (BMI) records were documented. A detailed history and clinical evaluation was performed. Skin thickness was evaluated by palpation and pinching of the face, upper arms, forearms and hands. Capillaroscopy was not conducted for lack of requisite equipment. The respiratory system was assessed using a detailed history, the Asthma Control Test (ACT), lung function testing and both skin prick testing and immunoblot assays to common aeroallergens. Similarly, suspected food allergy was excluded based on the history of exposure and relevant testing.

### Autoantibody Determination

#### Line Immunoblot Assay

Patient sera were analysed using the SSc [Nucleoli] Profile EuroLine [IgG]; Euroimmun, Catalogue Number DL 1532-1601G, a fixed immunoblot panel containing 13 autoantigens that detect SSc specific IgG autoantibodies. The 13 immunoblot panel included ATA, CENP-A, CENP-B, RP11, RP155, NOR 90, fibrillarin (or anti-U3RNP), Th/To, PM-Scl100, PMScl-75, Ku, PDGFR and Ro-52. The patients had been screened using the Ig G antibodies against nuclear antigens (ANA) immunoblot panel from the same manufacturer (Euroline Cat # DL 1590-6401-3G) that includes ATA, CENPB, PM-Scl100 and Ro-52 as well as non-SSc specific AMA-M2, SS-A, SS-B, dsDNA, nucleosomes, Sm, RNP/Sm, PCNA, histones, Rib-P-protein and Jo1. The patients were tested using either the SSc profile or the ANA profile. In some cases samples were tested with both panels. Testing was in accordance with the manufacturer’s instructions. Briefly, immunoblot test strips impregnated with different ENA specificities were incubated with a dilution of 15 microliters of serum in 1.5 ml (1:101) of sample buffer. Autoreactivity was detected by incubating the strips with alkaline phosphatase labelled anti-human IgG antisera. The addition of the NBT/BCIP substrate elicited a color reaction, that was evaluated using the manufacturer’s software (EUROlabScan) to report positive, borderline or negative reactivity. Positive control bands are included in each strip.

#### Immunofluorescence

Immunofluorescence examination was conducted in 16% of patients with the aim of correlating this standard method of SSc diagnosis with the line blot immunoassays. Patient sera were stained using the IIFT mosaic basic profile 3A slides (Cat. FA 1802-2010-3) and examined using the EuroStar III Plus immunofluorescence microscope (Euroimmun Ag, Germany). Reagents were from the same manufacturer.

#### Clinical Laboratory Measurements

Other laboratory investigations were requested as indicated in the course of routine clinical care, and not all 240 patients had all the tests. The parameters measured included but were not limited to complete blood cell counts, erythrocyte sedimentation rate (ESR), C-Reactive Protein (CRP), urinalysis, electrolytes, urea and creatinine, liver enzymes, Complement C3, C4, creatinine kinase (CK), serum IgG, IgA, IgM and IgE immunoglobulin levels, T lymphocyte subsets and thyroid function tests (TSH, T3, T4). Laboratory testing was conducted in an ISO 15189 accredited in-house clinical laboratory. T lymphocyte subset enumeration was performed using a FACSCalibur (Becton Dickinson) dual laser, four colour flow cytometer. Thyroid function parameters were measured using the ELISA technique, different commercial kits were used in the study period.

### Statistical Analysis

The one-way ANOVA test was used to compare the means in the different subgroups. The null hypothesis was that the means of the different variables was the same for the different groups of data. A p-value <0.05 was considered significant. The StatPlus (version 6.1) software was used.

### Ethics Statement

Institutional approval was obtained and ethical approval to conduct the study was granted by the Medical Research Council of Zimbabwe (MRCZ/B/1479).

## Results

### Patient Demographic Profile, Ages Gender, and Race

During the period 2013-2018, 4335 patients were referred to the Clinic. Amongst these, 240 had detectable SSc specific autoantibodies and were selected for further investigation. The age distribution of the population was bimodal, 20% were under the age of 16 years, mean age 7.56 (95% CI 2.73-12.38) years. The mean age of older patients was 41.93 (95% CI 39.79-44.07) years. The racial groupings were Black Africans (84.6%), White (11.3%) and Asians (4.1%). The ancestry of Blacks was Zimbabwean, Whites were European, mainly British and Asians were of the Indian subcontinent. The median age was 36 years, when stratified into racial subgroups, the median age was lowest in Asians (31.5 years), while Black (35 years) were younger than White patients (46 yrs.). Autoantibody positive patients <16 years were Black (45/48) or Asian (2/48), one was White. There was a female predominance amongst the adults, the Female to Male (F:M) was 3.5:1. In the <16-year age group the gender proportion was F:M= 1.2:1.

### Vital Signs and Baseline Laboratory Findings

#### Blood Pressure (BP)

The mean systolic BP for all the groups was normal, 121.66 (95% CI 114.76-128.56) mmHg. There were no significant differences between the autoantibody subgroups. Although mean diastolic BP readings were also normal 75.37 (CI 71.06-79.68) mmHg, there were significant differences between autoantibody subgroups, being higher in the anti-Th/To (78 mmHg, 95%CI=76-81) than the anti-CENPB (72 mmHg 95%CI=68-76 mmHg) groups. (p=0.015). The mean BP readings amongst three race groups were comparable to the overall means in all autoantibody subgroups.

### Laboratory Investigations

#### Haematology

Although mean hematology parameters were within normal reference ranges, there were variations that depended on the types of autoantibodies detected. The hemoglobin (Hb) concentration was higher in anti-RP11 (14.01 g/L, 95% CI=13.6-14.4 g/L) compared to the anti-RP155 (13.4g/L, 95%CI=13.1-13.6) (p=0.016) anti-CENPB (13.15, 95%CI=12.7-13.6 g/L) p=0.006, and anti-ATA (13.1 g/L, 95%CI= 12.4-13.9), p=0.019 positive patients.

The overall erythrocyte sedimentation rate (ESR) was elevated (50.6 mm/h) when all patients were pooled. The ESR however varied with the types of autoantibodies and was significantly higher in anti-CENPB (56.58, mm/h, 95% CI=50.8-62) than anti-NOR90 (41.9 mm/h 95% CI=32.9-50.8), p=0.008 and anti-Ku (41.96 mm/h, 95%CI=33.7-50.2 mm/h), p=0.005 groups. The C-Reactive protein (CRP) was positive in 20% of the patients.

#### Lymphocyte Subsets

The median total lymphocyte percentage was 35.37% (95%CI=31.91-38.42) with no significant inter-group variations. The mean absolute and percentage CD4+ and CD8+ T lymphocyte counts, while within normal ranges also varied with autoantibody types. The CD4+ T lymphocyte percentage was highest in the anti-ATA (47.33%, 95% CI= 41.97-52.70) and lowest in the anti-CENPB (41.72% CI 37.94-45.50) and anti-RP11 (41.56%, 95%CI 36.98-46.146) antibody positive patients. The mean CD8+ T lymphocyte percentage was 27.56%, (95% CI=22.79-32.66), being highest in the anti-CENPB (28.6%, 95%CI= 24.28-32.92) and lowest in the anti-ATA subgroups (23.7%, 95%CI=19.00-28.41). Values for the other subgroups were not statistically different.

#### Complement

Complement protein concentrations differed in tandem with SSc specific autoantibody types. Mean C3 complement values (0.126 g/L) were significantly higher in the anti-PMScl100 (p=0.017)), than the anti-CENPB or anti-RP155 groups (p=0.005).

The mean C4 complement levels were highest in the anti-ATA (0.045 g/L) and lowest (0.026 g/L) in the anti-CENPB positive subgroups (p=0.003).

#### Immunoglobin Isotypes

Four immunoglobulin isotypes (IgA, IgM, IgG and IgE) were measured. The quantities of total IgG and IgA serum antibodies varied depending on the SSc specific autoantibody profile.

Patients who were anti-ATA and anti-PMScl 100 autoantibody positive had the highest total IgG antibody levels. The IgG antibody concentrations were highest in the anti-ATA, 1.717 g/L (95% CI=1,316-2,118) and anti-PMScl 100, 1,581 g/L (95%CI= 1,310 1,851) positive patients. The values anti-fibrillarin (1.221 g/) and anti-Ku (1.217 g/L) anti-Ku, anti-PMScl751,310 g/L anti-RP155 (1,347 g/L) and anti-Th/To 1,301 g/L positive patients were significantly lower (**Table 2**).

**Table 2:** Comparison of total serum IgA, IgM, IgG antibody levels, complement C3, C4 and lymphocyte subset values in patients with different SSc-specific autoantibodies.

	Autoantibody	Mean g/L	95%Confidence Interval		p-value
**IgG antibody**	**PMScl-100**	**1,581**	**1,310**	**1,851**	
	a. Fibrillarin	1,221	1,129	1,313	0.001
	b. Ku	1,217	1,023	1,411	0.008
	c. NOR90	1,276	1,071	1,480	0.017
	d. PMScl-75	1,310	1,220	1,401	0.009
	e. Th/To	1,301	1,196	1,407	0.003
	**ATA (ATA)**	**1,717**	**1,316**	**2,118**	
	a. Fibrillarin	1,221	1,129	1,313	0.005
	b. Ku	1,217	1,023	1,411	0.002
	c. NOR90	1,276	1,071	1,480	0.004
	d. PMScl-75	1,310	1,220	1,401	0.002
	e. RP155	1,347	1,269	1,425	0.003
	f. Th/To	1,301	1,196	1,407	0.001
**IgA antibody**	**Fibrillarin**	**0.335**	**0.267**	**0.404**	0.005
	a. PMScl-75	0.234	0.195	0.273	
	**ATA**	**0.336**	**0.270**	**0.401**	0.005
	a. PMScl-75	0.234	0.195	0.273	
	**Ro-52**	**0.316**	**0.268**	**0.365**	0.013
	a. PMScl-75	0.234	0.195	0.273	
**Complement (C3)**		0.126 g/L.			
	**PMScl 100**	**0.136**	**0.089**	**0.183**	0.017
	a. CENPB	0.128	0.111	0.145	
	**Th/To**	**0.136**	**0.119**	**0.153**	0.023
	a. Ro-52	0.109	0.092	0.126	
**Complement C4**					
	**ATA**	0.045 g/L			0.003
	a. CENPB	0.026 g/L			
**Total Lymphocyte %**		35.37%	31.91	38.42	
**CD4+ T lymphocytes %**		%			
	**ATA**	**47.33**	**41.96**	**52.70**	
	a. anti-RP11	41.56	36.98	46.15	
	b. CENPB	41.72	37.94	45.50	
	**Th/To**	**45.45**	**42.47**	**48.43**	
	a. CENPB	41.72	37.94	45.50	
**CD8+ T lymphocytes %**		27.56%	22.79	32.66	
	**CENPB**	**28.6**	**24.28**	**32.92**	
	a. ATA	23.7	19.00	24.41	

The levels of IgA, IgG, complement and CD4+ T lymphocytes were highest in anti-ATA and anti-PMScl100 seropositive patients. The bold values were used as a comparator in the statistical analyses. The p-values are a comparison of the frequency of autoantibody detection relative to the bold values.

The mean IgA concentration for all the patients was 0.270 g/L. There were inter-group variations and the mean concentration of IgA was highest in the anti-ATA, (0.335 g/L) anti-fibrillarin (0.335 g/L) and anti-Ro52 (0.316 g/L) positive patients. The lowest values were obtained in anti-PMScl75 (0.234 g/L) positive patients (**Table 2**). IgM levels were comparable across the autoantibody subgroups. The mean IgE antibody levels of 164 kU/L (95% CI= -127-455) was comparable across all autoantibody subgroups.

#### Thyroid Function

Abnormal thyroid function (hypothyroidism or hyperthyroidism) was the commonest endocrine abnormalities occurring in 7% (17/240). Diabetes was diagnosed in 5% and parathyroid adenomas in three.

Thyroid hormone levels varied with types of autoantibodies.

Mean free thyroxine (fT4) levels for all the patients were 2.53 ng/dL. The concentrations varied with the types of SSc specific autoantibodies. The concentration of fT4 was highest in anti-ATA (3.36 ng/dL) and anti-PMScl100 (3.02ng/dL) positive patients. These values were significantly higher than in anti-fibrillarin (1.51 ng/dL), anti-Ku (1.05 ng/dL), anti-PMScl 75 (1.23 ng/dL) and anti-Ro-52 (1.32 ng/dL) positive patients (**Table 3**).

**Table 3:** Immunofluorescence staining patterns and immunoblot reactivity clinical characteristics of patients fulfilling the ACR/EULAR criteria for the diagnosis of systemic sclerosis.

Case Number	Lab ref	Gender, Age	Immunofluorescence staining pattern	Immunoblot result	Clinical classification
10695	5016	Female, 54 yrs.	positive	RP11, PM-Scl75	overlap
		Female, 35 yrs.	cytoplasmic, nucleolar,	RNAP 155	Diffuse
17170	4139	Female, 39 yrs.	positive, nucleolar use slide	Not done	diffuse
9818	2620	Male, 51 yrs.	speckled,	CENPB, RP11, RP155	limited
10101	5329	Female, 39 yrs.	positive, nucleolar, titer 1:1280. use slide	fibrillarin, AMA-M2, Ro-52, SS-A	Diffuse
12193	7208, 7715	Female, 45 yrs.	Positive, nucleolar	RP11, Th/To	limited
12634	5016	Female, 60 yrs.	Granular, speckled	RP11, RP155, PMScl 75,	overlap
13271	112974	Female, 45yrs	speckled, titer 1:640	Ro-52	limited
13908	289/114361	Male, 55 yrs.	Positive, nucleolar	CENPB, Th/To, PMScl 100	limited
17132	4973	Female, 42 yrs.	homogenous, positive	Scl-70, CENPB Neg	limited
17320	4648	Male 47 yrs.	Positive, speckled	CENP-B	limited
23190	11204	Female, 35 yrs.	nucleolar staining, Titer, 1:960	RNAP 155, Fibrillarin, NOR90, SSA	Diffuse

The mean free triiodothyronine (fT3) levels were 2.65 pg/mL. The highest fT3 concentrations were in anti-PMScl 100 positive patients (3.25 pg/mL, 95% CI 1.79-4.70). The lowest fT3 concentrations were in anti-PMScl75 positive patients. (1.99 pg/mL, CI 1.61-42.37) (p=0.02).

There were no statistically significant differences in the levels of Thyroid Stimulating Hormone (TSH) between the groups. The mean TSH levels were 2.58 mIU/mL (95% CI 1.22-4.03).

### Detection of SSc Specific Autoantibodies

#### Line Immunoblot Assays

**Figure 1:**
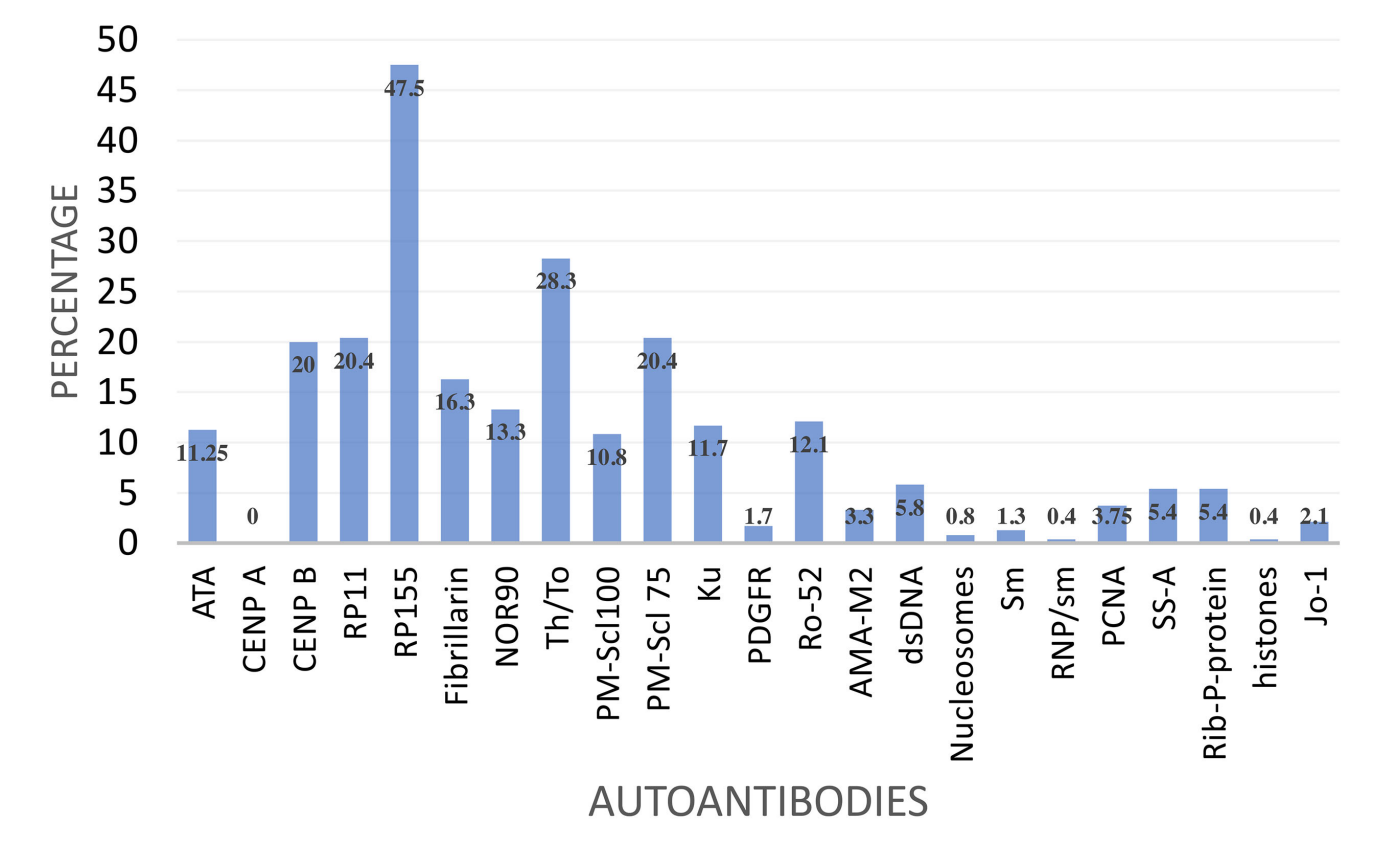
Frequency of reactivity to a panel of selected autoantibodies by SSc specific autoantibody reactive patients.

The two immunoblot panels that include SSc specific and associated autoantibodies were used. None of the patients (0%) had detectable anti-CENP-A. The remaining detectable autoantibodies were specific for anti-RNAP III 155 kD (RP155) 54.6%, anti-RNAP III, 11 kD (RP11) 21.6%. Other autoantibodies were anti-Th/To (27.9%) anti-CENPB (18%), anti-ATA/ATA (13%) anti-PMScl75 (17.9%), anti-NOR90 (12%) and anti-PMScl100 (10.4%). The SSc associated anti-Ku antibody was detected in 12% (**Figure 1**). There was infrequent reactivity to SLE specific autoantibodies anti-dsDNA (5.8%), anti-Rib-P-protein (5.4%) and anti-PCNA (4%). The Sjogren’s syndrome associated anti-SS-A was detected in 5.4% while the inflammatory myopathy associated anti-Jo-1 was detected in 2.1%.

### Immunofluorescence Reactivity

Reactivity to autoantigens was investigated by immunofluorescent (IF) microscopy of Hep-2 cells. The characteristic speckled, threads, nucleolar and centromere staining patterns were observed in some of the patients. Staining patterns that could not be categorized were classified as positive IFT.

Eleven (4.6%) fulfilled the ACR/EULAR criteria for SSc. Six had lcSSc, three dcSSc and three had symptoms suggestive of an overlap between SSc and inflammatory myopathies. One, RNAP 155, fibrillarin, NOR90 and SS-A positive female patient with rapidly progressive dcSSc succumbed, aged 35 years, within 15 months of the diagnosis of SSc. Results for the eleven ACR/EULAR consistent patients are presented in **Table 3**.

### Co-Occurrence of the Major Autoantibodies

The co-occurrence of autoantibodies was common, however the expression of (i) anti-ATA and anti-CENPB and (ii) anti-ATA and anti-RNAP11 was mutually exclusive. anti-RNAP155 and anti-ATA were co-associated in 4/31 (13%), anti-CENP-B and anti-RNAP155 in 8/44 (18%) and anti-RNAP11 and anti-CENPB in 5/44 (11%) patients. The RNA polymerase III autoantibodies were infrequently co-associated, anti-RP11 and anti-RP155 were co-associated in 12.9% (31/240). The two PMScl exosomes, (PMScl-100 and PMScl-75) were only co-associated in 3/61 (4.9%).

### Autoantibodies in Different Racial Groups

**Figure 2:**
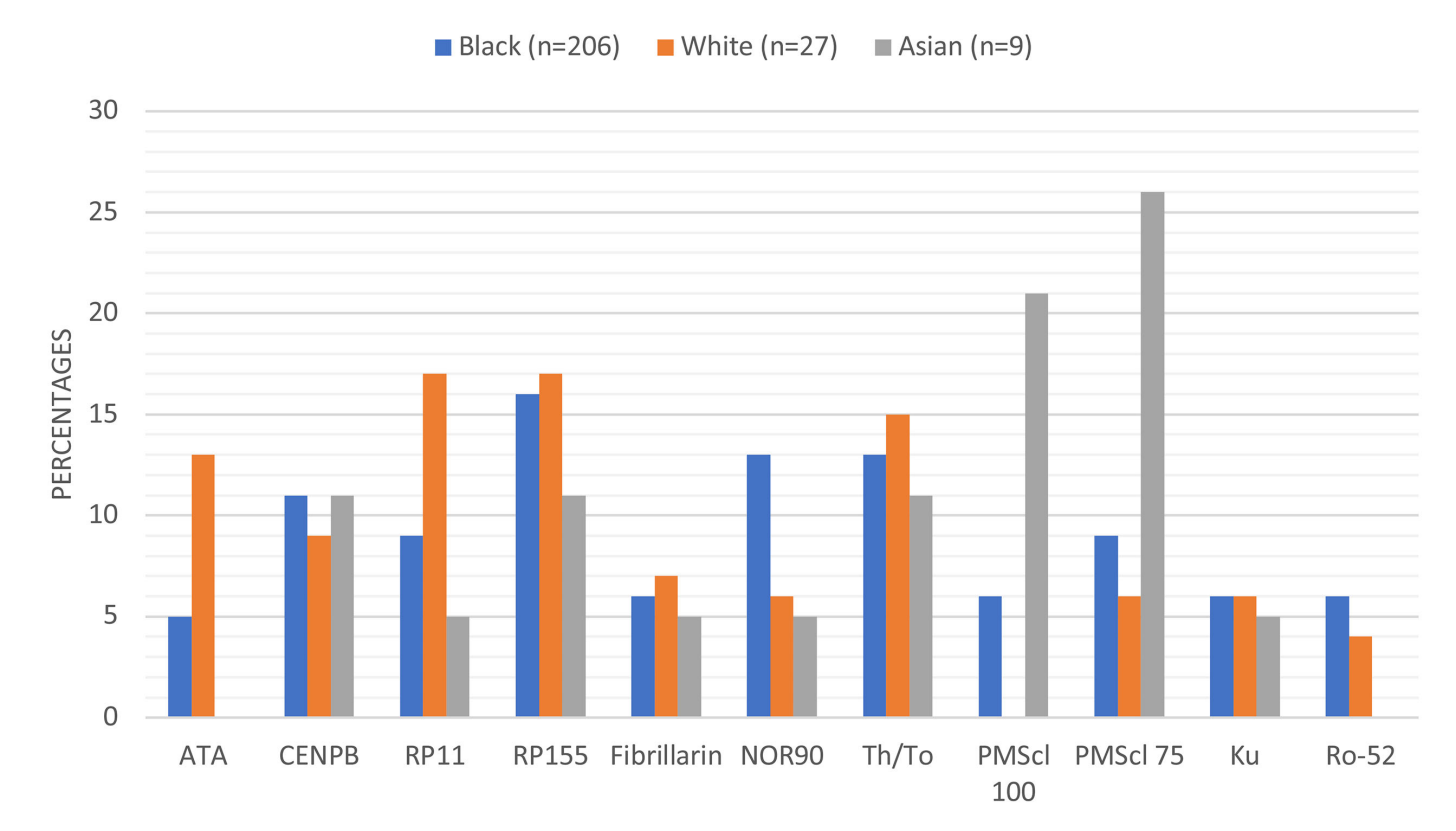
Proportions and types of autoantibodies detected in different racial groups. Anti-ATA and anti-Ro-52 were not detected in any of the Asian patients and anti-PMScl-100 was not detected in any White patient.

There were racial differences in the frequency of autoantibody detection (**Figure 2**). Anti-ATA was detected in 48% (13/27) White compared to 5% (10/203) Blacks and 0% Asians. Anti-RNAP 11 detection was highest in White (17%) compared to Black (9%) or Asian (5%) patients. Anti-NOR90 was twice more frequent in Black (13%) than in White (6%) or Asian (5%) patients. Anti-PMScl100 and anti-PMScl75 were more frequent in Asian patients. The detection rate for anti-CENPB, anti-RP155, anti-Th/To and anti-Ku was comparable across the racial subgroups. The most frequently detected autoantibodies amongst the 27 White patients were anti-RP11, anti-RP155, anti-Th/To, anti-ATA and anti-CENP-B. None of the White patients had detectable anti-PM-Scl-100 antibodies.

### Organ Involvement

The symptoms were referrable to multiple organs predominantly the respiratory (92%), cutaneous (87%), musculoskeletal (51%) and gastrointestinal (47%). Symptoms of Raynaud’s vasculopathy were reported by 24% of the patients. The type and frequency of involvement of different organs is summarized in **Table 4**.

**Table 4:** Frequency and types of vascular, respiratory, gastrointestinal, cutaneous and musculoskeletal symptoms reported by patients with SSc-specific autoantibodies.

Raynaud’s Phenomenon	Raynaud’s Vasculopathy	N	%
		57	24%
Respiratory symptoms			
	cough	174	75.5%
	Dyspnea/tightness of the chest	162	67%
	wheeze	59	24.60%
	nasal discharge	59	24.6%
	sneezing	54	22.50%
	Nasal blockage	47	19.60%
	asthma	42	17.50%
	chest pain	18	7.50%
	Rhinorrhea	37	15.4%
	Posterior nasal drip	34	14%
	Allergic rhinitis	27	11.5%
Gastrointestinal			
	constipation	114	47.5%
	Abdominal pain	113	47%
	bloating	84	35%
	Gastro-esophageal Reflux	72	30%
	Heartburn	68	28.3%
	Dysphagia/esophageal dysmotility	42	17.5%
	Peptic ulcers	38	15.8%
	Vomiting	37	15.4%
	Diarrhea	26	10.8%
	Inflammatory bowel disease/syndrome	23	9.6%
Cutaneous			
	urticaria and pruritus	195	81%
	Skin hypo/hyperpigmentation	51	21.3%
	Alopecia or hair thinning	40	16.7%
	psoriasiform rash (morphea)?	37	15%
	Alopecia or hair thinning	40	16.7%
	eczema	39	16.3%
	perioral or periorbital hyperpigmentation	12	5%
	cutaneous blisters or skin ulcers	11	4.6%
	Classical morphoea	7	2.9%
	Calcinosis, teleangiectasia, skin keratinization	8	4.5%
	fingertip ulcers	5	2.1%
Tissue swelling			
	Angioedema (19) face (23), lips (17), eyelids (16)	19	12%
	Hands or feet (including palms and soles)	34	14%
	Puffy fingers	24	10%
	Generalized swelling	10	4.2%
	Other swelling Ear lobe (5), scalp (3), torso (2)	11	4.6%
Musculoskeletal			
	joint pain unspecified (93), knee (57), finger (41), ankle (24), wrist (20), hip (16), feet (12), elbow (12)	93	39%
	Muscle cramps	13	5.4%
	Madonna fingers	3	1.3%
	Jaw movement limitation	3	1.25%

### Respiratory Tract

Respiratory symptoms were dominant (92%) and included dyspnea, a non-productive dry cough, wheezing, chest pain and chest tightness (6%). Bi-basal velcro sounds were often heard on auscultation. The Asthma Control Test (ACT), a subjective symptom assessment tool gave an average score of 16 (range 6 to 25) amongst those with respiratory symptoms. Nebulization with beta-2 agonist bronchodilators infrequently demonstrated significant changes in FVC, FEV1 or FEF25-75. The values were often depressed following bronchodilator inhalation. Bronchodilator reversibility was only observed in a subset of patients with a positive family history of atopy, reactivity to seasonally relevant inhalant allergens and a clinical diagnosis of asthma (17.5%) and/or allergic rhinitis (11.5%). Pulmonary function parameters varied with the types of autoantibodies detected (**Table 5**).

**Table 5:** Variation of pulmonary function test results FEV1 and FVC depending on the types of serum autoantibodies.

Lung Function Test		*Mean*	*95% Confidence interval*		*p-value*
FEV_1_					
	*PMScl-100*	*87.7*	*79.2*	*96.2*	
	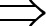 PMScl-75	73.6	65.5	81.6	0.017
	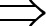 Ku	70.9	61.3	80.5	0.011
	Th/To	85.1	78.9	91.4	
	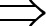 PMScl-75	73.6	65.5	81.6	0.017
	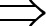 Ku	70.9	61.3	80.5	0.011
FVC					
	PMScl 100	82.352	73.2	91.5	
	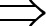 Ro-52	69.8	64.9	74.8	0.019
	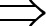 PMScl 75	68.7	61	76.4	0.014
	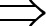 Ku	65.7	56.4	75	0.007
					
	Th/To	78.7	72.7	84.6	0.013
	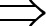 Ku	65.7	56.4	75	
FEV1/FVC ratio		%			
	Ro-52	112	108.6	115.4	
	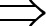 NOR90	104.9	99.79	109.9	0.02
	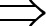 RP155	106.5	104.1	108.8	0.01
	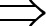 Th/To	106.8	103.8	109.7	0.02
PEFR					
	PMScl 75	107.6	103.3	111.8	
	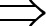 §	89.7	78.7	100.8	0.006
	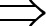 Ro	82.7	74	91.5	0.001
	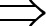 ATA	81.2	62.4	100.1	0.003
	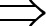 NOR90	77.6	62.6	92.6	0.001
	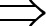 PMScl100	76	67.5	84.5	0.0001
	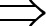 RP155	75	69.4	80.6	0.002
	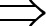 RP11	74	63.4	84.6	0.018
	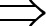 Ku	73.8	64.7	82.9	0.0001
	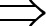 Fibrillarin	71.9	63.5	80.4	0.009

Patients with detectable anti-PMScl-100 and anti-Th/To had significantly higher FEV_1_ and FVC values than those who were anti-PMScl-75, anti-Ku or anti-Ro-52 positive. The highest PEFR values were recorded in anti-PMScl75 positive patients.

The FEV_1_ varied with the types of detectable autoantibodies. The mean FEV _1_ for all the patients (79.96%) was in the lower limit of normal values. The highest FEV1 volumes were documented in anti-PMScl-100 (87.7%) and anti-Th/To (85.1%) autoantibody positive patients. The FEV1 values for the anti-PMScl-75 (73.6%) p=0.017 and anti-Ku (70.9%) positive patients were significantly lower than the group means and the anti-PMScl100 values.

The FVC values were decreased (mean 74%).

The FVC values varied with the types of detectable SSc specific autoantibodies. FVC% values for anti-PMScl100 (82.35%, CI 73.17-91.53) and anti-Th/To (78.66%, CI 72.69-84.63) positive patients were within were within normal (80-120%) or near-normal ranges and higher than the group means. In contrast the FVC% values for the anti-Ku (65.7%), p=0.013, anti-PMScl75 (68.7%) (p=0.014) and anti-Ro52 (69.86%) positive patients were significantly lower than the normal ranges.

The mean FEV1/FVC ratio for all the patients combined was (107.6%, 95%CI 104.19-111.91) significantly greater than predicted for age, gender, ethnicity, and BMI suggesting a predominantly restrictive pulmonary defect. The severity of the restrictive changes varied with the types of autoantibodies detected. The highest was FEV_1_/FVC ratios were in anti-Ro-52 (112; 108.61-115.39), anti-CENPB (111.07, CI=106.37-115.77) and anti-Ku (109.19, CI=105.70-112.68) positive patients. High FEV_1_/FVC ratios were also documented in anti-NOR90 (105%), anti-RP155 (106.5%) and anti-Th/To (108.8%) positive patients. When the FEV_1_/FVC ratio of the non-SSc specific anti-Ro-52 antibody positive patients was compared with that of patients with the SSc specific anti-NOR90, anti-RNAP155 and anti-Th/To groups the differences were significant (p=0.01).

The mean PEFR for all the patients was 87% of predicted values. There were differences in PEFR values depending on detectable autoantibodies. The mean PEFR of 107.6% for the anti-PMScl-75 positive patients was significantly higher than in the anti-Th/To (89.7%), p=0.006.

### Skin Involvement and Tissue Swelling

**Figure 3:**
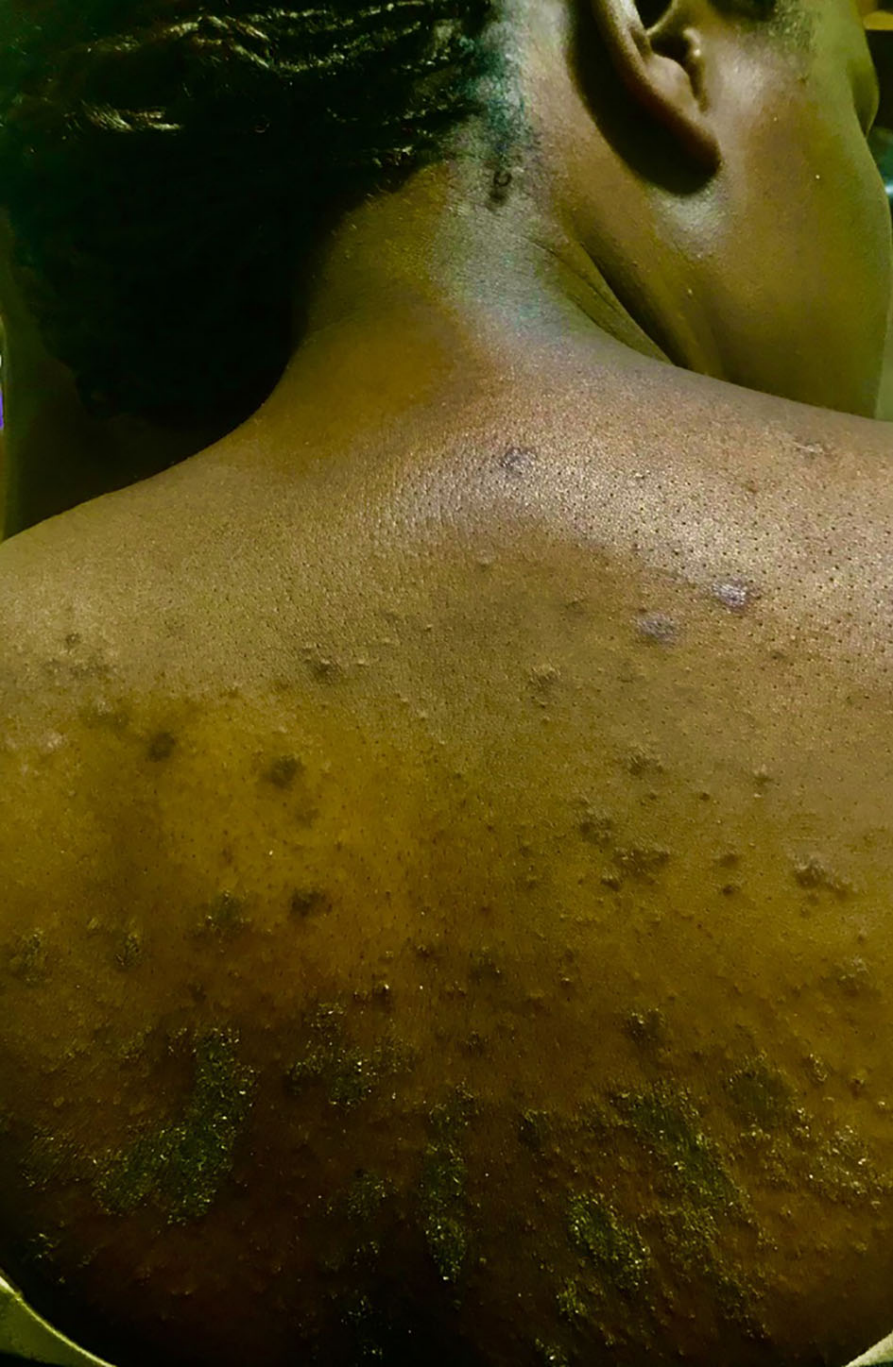
Psoriasiform dermatosis in a patients with SSc specific autoantibodies.

**Figure 4:**
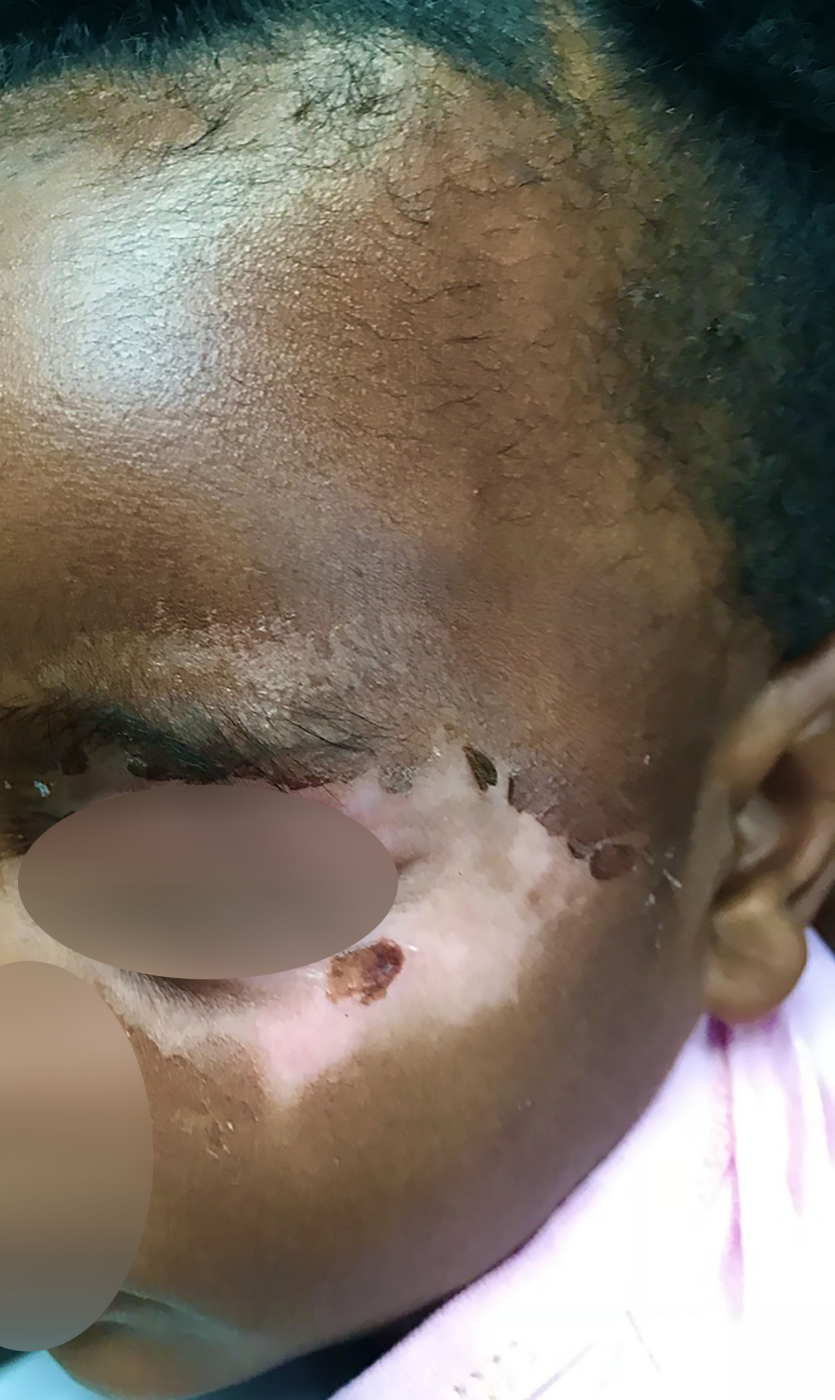
Figure shows skin changes and alopecia in a child with detectable SSc specific autoantibodies.

Cutaneous symptoms were present in 87% (209/240) of patients in all age groups. The most frequent was a predominantly acral pruritic rash of different morphological descriptions (76%), urticaria (38%), dermatitis/eczema (30%), hypo/hyperpigmentation (23%) and a psoriasiform rash (7%) (**Figure 3**). The pruritus was often antihistamine refractory. Morphoea and various dermatoses including a pruritic rash with a psoriasiform morphology were frequent findings in patients of all ages including a 2-year-old (**Figure 4**). Hair thinning, hair loss and alopecia were common (17%) (**Figure 4**). Keratinization, digital ulceration, sclerodactyly and calcinosis were infrequent, being observed in the subgroup that fulfilled the ARA/EULAR criteria for SSc.

**Figure 5:**
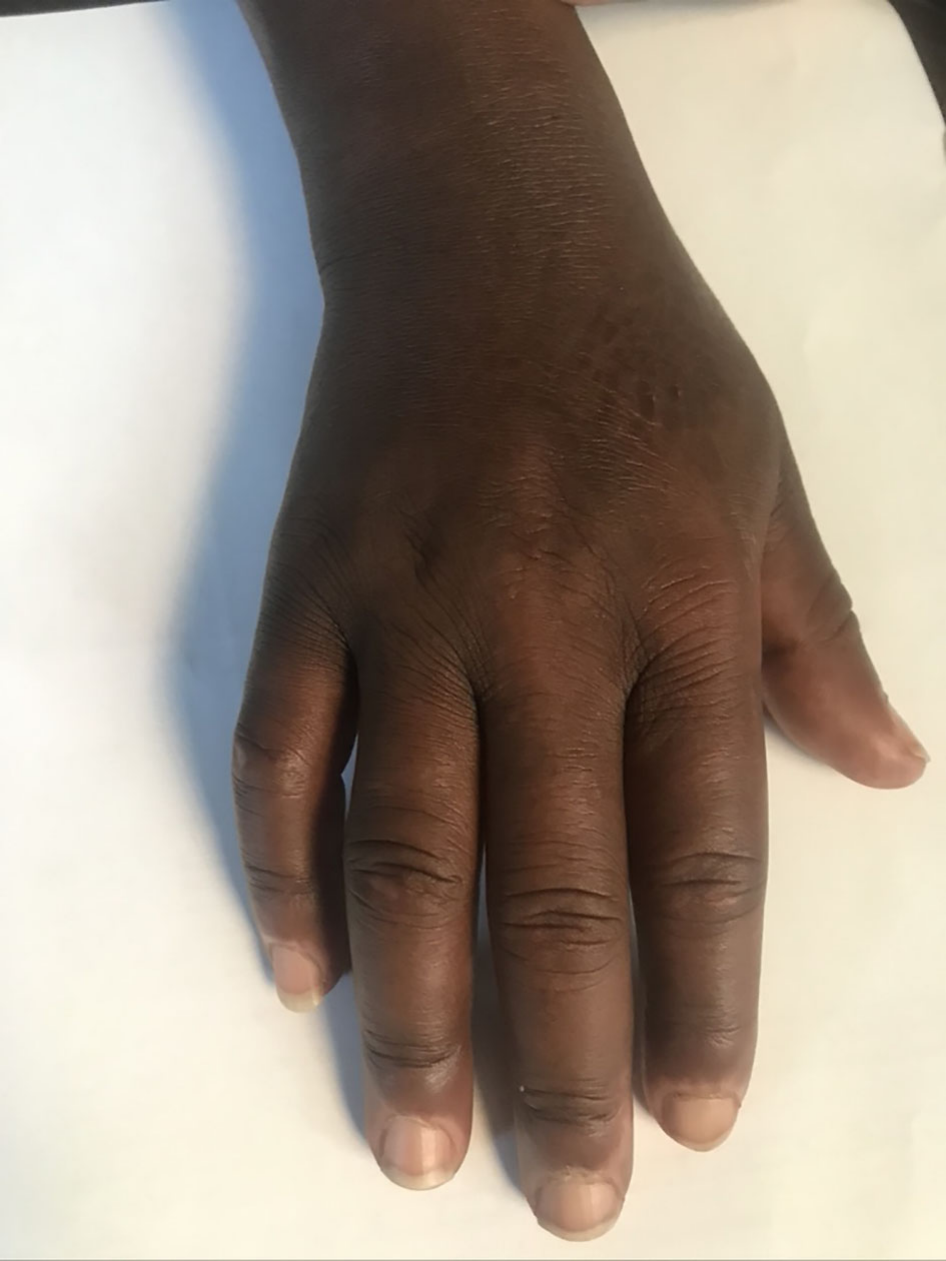
Puffy fingers and cuticle hypo pigmentation in a child with detectable SSc specific autoantibodies.

Subcutaneous tissue swelling was observed in 41% (99/240). This included angioedema (12%) variously involving the lips, eye lids or hands (6.7%). Skin thickening was observed in 5% of the patients. Puffy fingers (**Figure 5**) were observed in 5% and were most prominent in pediatric groups.

### Gastrointestinal Tract

The most frequent gastrointestinal symptoms were nausea (51%), constipation (48%) abdominal and/or epigastric pain (47%), bloating (35%) and gastro-esophageal reflux (30%) and constipation. The symptoms were associated with the presence of defined SSc specific autoantibodies. Constipation was associated with anti-ATA (p=0.033), anti-NOR90 (p=0.039) and anti-Ku (p=0.026). Diarrhea was associated with anti-ATA (p=0.001) and anti-PM-Scl75 (p=0.005), abdominal pain with anti-RP 155 (p=0.023), GERD with anti-fibrillarin (p=0.011) and bloating with anti-Th/To (p=0.011). The most common symptom, nausea and other poorly localizable symptoms (e.g. heartburn, belching, dyspepsia and melena) showed no significant associations with specific autoantibodies.

Musculoskeletal involvement was documented in 51% (122/240) of the patients. Joint pains (39%) involving knees (24%), fingers (17%), ankles (10%) and wrists (8%) were dominant. Muscle cramps were reported by 5%. Madonna fingers were seen in three patients. Neurological and psychiatric conditions were reported by 54% (130/240) with headaches (31%) being dominant. The carpal tunnel syndrome was reported by 3%.

Ocular symptoms were reported in 46% (110/240) of the patients (**Supplementary Table 3**). These reported Itchy (35%), inflamed (25%) or red (18%) eyes. The ocular sensations were described as sand grain (13%), gritty (7%), dry (3%) or dry like sandpaper. Allergic conjunctivitis (7.5%) was diagnosed in a subgroup with a positive family history of atopy and SPT or immunoblot confirmed sensitization to aeroallergens. Eye discharge or sticking of the eyelids was in 6% and tearing in 4.5%. Photosensitivity and photophobia were commonly reported (**Supplementary Table 2**).

### Neurological Symptoms

The dominant neurological symptoms were headaches (31%), the carpal tunnel syndrome was infrequent (3%) and only diagnosed in patients with the dc-SSc associated autoantibodies.

Cardiovascular symptoms were infrequent, being reported in 42/240 (17.5%) of the patients (**Supplementary Table 3**). Retrosternal or pericardial pain (8%) palpitation (7%) and hypertension (3%) were dominant. Cardiomegaly, cardiomyopathy, pericardial effusion, chronic cardiac failure, and a history of myocardial infarction were diagnosed or reported in two patients each, while pulmonary embolism, arrythmia, ventricular septal defect and tricuspid regurgitation in one patient each.

The systemic, endocrine, gynecological, renal, hematological, immunological and malignant conditions detected in patients expressing SSc specific autoantibodies are summarized in **Supplementary Table 2**.

Raynaud’s disease (24%) was the dominant vascular abnormality. Lymphadenopathy was recorded in 7% of the patients. Non-specific systemic features included tiredness (7%), exhaustion, feeling drained, sweating, fever and chills. Menstrual irregularities were the most frequent gynecological conditions, one (28-year-old) reported premature menopause.

Renal diseases affected 7% of the patients, malignant hypertension being the most severe and the remainder being documented after urinalysis findings. Cortical kidney cysts were detected in two patients. Current or prior diagnoses of malignancy were documented in 2.5% (6/240) of the patients and involved cervical and breast cancer. Other malignant conditions were Kaposi’s sarcoma and non-Hodgkin’s lymphoma.

The co-existing autoimmune conditions were Sjogren’s syndrome (n=1), Myeloperoxidase (MPO) positive vasculitis (n=1) and myasthenia gravis (n=1). Patch test reactivity to epoxy resin, thiuram mix, potassium dichromate and paraben esters that was confirmed in four SSc-specific antibody positive patients were the only potential associations of SSc autoantibody production with an occupational allergen. Anemia and von Willebrand’s disease were the only hematological abnormalities. Current infection with tropical diseases was infrequent, two had *Schistosoma haematobium*.

## Discussion

This study describes the clinical and laboratory findings in symptomatic Zimbabwean patients who were referred for the evaluation of often non-specific skin, respiratory or gastrointestinal tract, ocular and musculoskeletal ailments. All were reactive to extractable SSc specific cytoplasmic or nuclear antigens. Although a majority of the patients did not fulfil ARA/EULAR criteria for SSc, their symptoms were consistent with those recognized in SSc. A sub-sample of the patients was offered immunofluorescence testing to investigate concordance with standard SSc laboratory diagnostic criteria. The results of the comparison were concordant. The autoantibody profiles differed with the racial groups. An association between the presence of autoantibodies and disease phenotypes was observed with respect to the respiratory and gastrointestinal tracts and thyroid abnormalities.

### Patients

The overall female predominance amongst adult patients (F:M=3.5:1) is comparable to other studies (17, 18). The median adult ages differed significantly along racial subgroups being lowest in Asians (31.5 years), Blacks (35 years) and highest in White (48 years) patients. This difference could explain the recognized earlier onset of SSc in Asian and Black patients. A remarkable finding of this study was that 20% of autoantibody positive patients were <16 years old (mean age 7.56, 95%CI 2.7-12). These were Black (45/48), Asian (2/48) and White (1/48). The racial differences suggest that the presumed rarity of SSc in children could be due to study biases that predominantly report findings in White but not Black patients. To our knowledge, this number of Black pediatric patients with symptoms suggestive of SSc and detectable SSc specific autoantibody reactivity has not been previously reported.

### Blood Pressure

Overall systolic and diastolic BP readings were within normal ranges. However, the diastolic pressure readings varied with the types of detected autoantibodies. Diastolic BP was highest in the anti-ATA group and lowest in the anti-PMScl 75 group. The differences in BP could suggest that the types of antibodies produced influence disease phenotypes, skewing patients to towards either the dcSSc or lcSSc phenotypes. The diastolic BP differences between two lcSSc associated anti-Th/To (78 mmHg, 95%CI=76-81) and anti-CENPB (72 mmHg 95%CI=68-76 mmHg) subgroups, (p=0.015) could indicate the recognized higher frequency of renal crisis in association with Th/To but not anti-CENPB as previously reported (19).

### Detection of SSc Specific Autoantibodies in Different Racial Subgroups

The detection of autoantibodies varied with race, anti-ATA and anti-RP11 were more frequently detected in White than Black patients, while anti-PM-Scl 100 was detected in 21% Asian and 6% Black and none (0%) White patients (**Table 3**). The frequency of the lsSSc associated anti-CENPB, anti-RP155, anti-Th/To and anti-Ku was comparable across racial groups. The racial variations that reflect genetic differences, suggest that autoantibodies influence disease phenotypes. The different types of autoantibodies detected in racial groups, makes the targeted tailoring of SSc test panels for defined population groups, one size does not fit all. The textbook focus on anti-ATA and anti-ACA and more recently anti-polymerase III antibodies as prime biomarkers for SSc likely underestimates the prevalence of SSc specific antibodies amongst Black patients, who overwhelmingly expressed anti-RNAP155 in this study.

#### SSc Diagnosis

The ARA/EULAR criteria for the diagnosis of SSc are internationally accepted. Although only 5%, fulfilled the ARA/EULAR criteria for SSc, all the patients had symptoms that are recognized in established SSc. Raynaud’s Phenomenon (RP) an early precursor and predictor of progression to overt SSc was documented in 24%. This figure may underestimate the true prevalence, since cutaneous manifestations of RP in African patients are not obvious and have to be pro-actively solicited. They are affected by the skin colour as well as the climatic conditions. Patients often reported cold hands or feet in colder months of the year. While it is recognized that the mere presence of SSc specific autoantibodies is *per se* not diagnostic of SSc, these are associated with early symptoms that predate overt SSc (5, 15). The purpose of making a diagnosis is to inform the clinical management of patients. In that vein, a combination of the clinical symptoms and the results of detailed physical examination, complemented by relevant laboratory or imaging test results should be prioritized over the fulfilment of international criteria when making a diagnosis. Nevertheless, due to the non-fulfilment of these standard criteria, we avoided labelling all patients as SSc, preferring to describe them as possible early SSc. Nail fold capillaroscopy, a key component for the fulfilment of the Le Roy and Medsger (20) and VEDOSS (21) criteria for early SSc could not be performed. Longitudinal follow-up studies to establish whether or not these patients have SSc and to monitor any disease progression particularly in the pediatric population are recommended.

### Laboratory Findings

Laboratory tests were requested and individualized for the routine clinical care of the patients, therefore patients did not have uniform panels of laboratory tests. The elevation of the inflammatory marker ESR, positivity of CRP in 20% and the presence of reactive lymphadenopathy (16%) indicates ongoing inflammatory activity in a proportion of this patient population. The presence and persistence of inflammation in SSc is recognized and has been previously reported (22, 23). Although the patients had GIT symptoms, anemia, a feature of advanced SSc was not observed (24). Anemia is considered less likely in early SSc. The higher mean IgE levels reported in this study could be explained by the inclusion of patients with co-existing allergic asthma and/or rhinitis.

#### Immune Responses

The innate, cellular, and humoral responses to SSc autoantigens varied significantly with the types of autoantibodies (**Table 6**). Higher levels of Complement (C3), IgA and IgG as well as CD4+ T lymphocytes were more frequent in patients with dc-SSc specific autoantibodies (ATA, PMScl 100 and anti-RP11) than in those with lc-SSc associated autoantibodies (CENPB, Ku and Th/To) who also had lower C3 complement, IgA, IgG antibody levels and CD4+ T lymphocyte percentages. The implication is that immune responses to target autoantigens influence disease phenotypes. Interestingly, the CD8+ lymphocyte percentages were, in contrast, higher in anti-CENPB than anti-ATA positive patients.

**Table 6:** Comparison of thyroxine (T3) and triiodothyronine (T4) hormone levels in patients with different types of autoantibodies.

Thyroxine (T4)		Normal range: 0.7-1.8 ng/dL	95% Confidence Interval		p-value
	**ATA**	**3.37**	**0.77**	**5.97**	
	a. fibrillarin	1.51	0.82	2.19	0.025
	b. Ku	1.05	0.79	1.32	0.024
	c. PMScl-75	1.23	1.04	1.43	0.010
	d. Ro-52	1.32	1.05	1.59	0.017
	**PMScl 100**	**3.02**	**1.07**	**4.97**	**0.022**
	PMScl75	1.23	1.04	1.43	
**Triiodothyronine (T3)**		***Normal range: 2.3 - 4.1 pg/mL***			
	**PMScl 100**	**3.25**	**1.79**	**4.71**	**0.020**
	PMScl 75	1.99	1.61	2.37	
**Thyroid Stimulating Hormone (TSH)**	Mean for all patients	2.58 mIU/mL	1.22	4.03	

Autoantibodies that are associated with diffuse cutaneous SSc (anti-ATA, anti-PMScl100) were associated with significantly higher hormone levels.

The bold values were used as a comparator in the statistical analyses. The p-values are a comparison of the frequency of autoantibody detection relative to the bold values.

#### Thyroid Function

The frequency of thyroid abnormalities in this cohort was higher than in the background population suggesting it could be attributable to the autoantibodies. Abnormalities of thyroid function varied with types of SSc autoantibodies. The dc-SSc associated autoantibodies (ATA and PMScl 100) correlated with higher levels of fT3 and fT4, while the lc-SSc associated autoantibodes correlated with lower fT3 and fT4 levels. An association between anti-ATA and thyroid dysfunction is recognized (25). There was a dichotomous relationship between the two PMScl autoantibodies and thyroid disease. Anti-PMScl 100 antibody positive patients had higher fT4 while anti-PMScl75 positive had lower fT3 levels suggesting different effects of the two PMScl antibodies on thyroid function.

### Cutaneous Disease

Skin involvement in early stages of SSc consists of different morphological features. The anatomical distribution of the dermatoses tended to be acral with limited or transient flexural involvement. Care providers should consider SSc as a differential diagnosis in managing these frequently atypical dermatoses. The dominant cutaneous symptoms were non-specific pruritus (54%), urticaria (28%) and dermatitis (10%) that are not considered hallmarks of SSc. The characteristic finger-tip ulcers, calcinosis, keratinization of the skin and the classic Madonna were infrequent, being observed amongst those fulfilling the ARA/EULAR criteria. Non-pitting edema of the hands or feet and puffy fingers were prominent in the younger patients.

### Respiratory Tract Disease

The predominance (92%) of patients with respiratory symptoms may have been influenced by a referral bias to the Asthma and Allergy Clinic. However, most of the autoantibody positive patients did not have allergic asthma or rhinitis. Lung volume abnormalities were bronchodilator refractory, and the spirometry pattern was predominantly restrictive, contrary to the obstructive, bronchodilator reversible pattern that characterizes asthma. Taken together, with findings of Velcro crackles on auscultation, the spirometry findings suggest fibrotic pulmonary disease, a feature of pulmonary SSc (18, 26). The significant association of the presence of anti-Ku, anti-PMScl-75 and anti-Ro-52 with FVC reductions supports the association of anti-Ku and anti-PMScl75 with ILD as reported by others (27, 28).

#### Gastrointestinal Tract

The significant association of constipation, diarrhea, abdominal pain, GERD and bloating respectively with anti-ATA, anti-NOR90, anti-Ku anti-PM-Scl75, anti-RP 155, anti-fibrillarin and anti-Th/To suggests that these autoantibodies influence the symptom manifestations. Since the frequency of the antibodies differs with racial groups, these could also explain racial differences in the types and severity of GIT symptoms.

### Neurological and Psychiatric Conditions

There was an association between autoantibodies with central and peripheral nervous system symptoms that are recognized in SSc. The carpal tunnel syndrome was only reported in anti-fibrillarin and anti-ATA positive patients. Psychiatric conditions were infrequent, anxiety featured prominently.

Cardiovascular was infrequent as expected in an early SSc cohort. Renal involvement was limited. There were no significant renal abnormalities and observed hematuria and kidney cysts could reflect their prevalence in the background population.

### Shortcomings

The main shortcoming is that this paper was not prospective and collected data were not standardized. The absence of a prospectively administered standardized questionnaire poses a risk under reporting the symptoms that were not solicited. In mitigation, all patients were attended to by one of us only (ES) who obtained case histories, examined all the patients and requested the laboratory and imaging tests. Therefore, notwithstanding the shortcomings, any inherent bias is therefore likely to be uniform. While the presence of antibodies suggests SSc, only 5% of the patients fulfilled ARA/EULAR criteria that are designed for the inclusion of patients in clinical trials. These criteria may not be appropriate for early SSc. We suspect our antibody positive symptomatic patients 24% of whom had RP could have early SSc that could not be confirmed or excluded for lack of requisite equipment. The study describes the clinical presentations and laboratory findings that included SSc specific autoantibody reactivity who may or may not have SSc. The lack of access to nail-fold capillaroscopy limited our ability to confirm early SSc, longitudinal studies if funded will help answer that question.

## Conclusion

This report covers the clinical history, clinical findings and laboratory investigations conducted on 240 Zimbabwean patients with detectable SSc specific antibodies, 20% of them being younger than 16 years. The observed cutaneous, respiratory, gastrointestinal, musculoskeletal and endocrine aberrations are consistent with findings reported in patients fulfilling the ARA/EULAR criteria. A tendency of alignment of respiratory, gastrointestinal, laboratory and endocrine findings with either dc-SSc or lc-SSc specific autoantibodies was observed. The findings suggest a decisive influence of innate, humoral and adaptive immune responses on disease phenotypes. Racial differences in autoantibody presence and the dominance of anti-RNAP antibodies amongst Black patients, should inform the tailoring of autoantibody test panels to specific populations. We conclude that the presence of SSc specific autoantibodies is common, albeit infrequently detected in Zimbabwe. A fifth of our patients were under 16 years old and presented with non-specific symptoms that should be investigated with a higher index of suspicion in this and similar settings.

## Data Availability Statement

The raw data supporting the conclusions of this article will be made available by the authors, without undue reservation.

## Ethics Statement

The studies involving human participants were reviewed and approved by Medical Research Council of Zimbabwe (MRCZ). Written informed consent to participate in this study was provided by the participant’s legal guardian/next of kin.

## Author Contributions

ES formulated the research idea, performed clinical evaluation of all the patients, supervised the laboratory testing, reviewed and revised the manuscript. YD wrote the initial draft of the manuscript, searched for all the references. MC participated in data collection. TM critically reviewed the manuscript and approved submission. FM critically reviewed the submission of the manuscript. All authors contributed to the article and approved the submitted version.

## Funding

This research was supported by the OAK Foundation studentship grants. This research is also commissioned by the National Institute for Health Research (NIHR), using Official Development Assistance (ODA) funding 16/136/33. The views expressed in this publication are those of the authors and not necessarily those of the NIHR or the Department of Health. The funders had no role in the conception, study design, data collection and analysis, decision to publish or preparation of the manuscript.

## Conflict of Interest

The authors declare that the research was conducted in the absence of any commercial or financial relationships that could be construed as a potential conflict of interest.

## Publisher’s Note

All claims expressed in this article are solely those of the authors and do not necessarily represent those of their affiliated organizations, or those of the publisher, the editors and the reviewers. Any product that may be evaluated in this article, or claim that may be made by its manufacturer, is not guaranteed or endorsed by the publisher.

## Supplementary Material

Supplementary Table 1 Blood Pressure measurement.

Supplementary Table 2 Comparison total Immunoglobulin IgA, IgG, IgM concentrations in patients with different SSc specific autoantibodies.
