# Supplementary material for: Systemic Sclerosis in Zimbabwe: Autoantibody Biomarkers, Clinical, and Laboratory Correlates
Source: Front Immunol. 2021 Nov 9;12:679531. doi: 10.3389/fimmu.2021.679531 (PMC8631108; doi:10.3389/fimmu.2021.679531)
Supplement: Supplementary Table 1 — Blood Pressure measurement. [file Table_1.docx]

Supplementary Table 1

Systolic and diastolic blood pressure measurements segregated according to autoantibodies expressed.

|  | **Systolic (range)** | **Diastolic (Range)** |
| --- | --- | --- |
| Scl-70 | *125 (91-150)* | *73 (59-90)* |
| CENPB | *120 (86-177)* | *72 (51-87)* |
| RNAP155 | *122(86-170)* | *75 (51-103)* |
| RNAP 11 | *124 (101-160)* | *75 (54-100)* |
| NOR90 | *124 (86-150)* | *74 (51-93)* |
| Th/To | *122 (86-162)* | *78 (51-97)* |
| PMScl 100 | *124 (101-154)* | *77 (64-97)* |
| PMScl 75 | *115 (86-150)* | *73 (51-93)* |
| Ku | *122 (86-151)* | *74 (93-121)* |
| Ro-52 | *122 (102-150)* | *74 (60-94)* |
| Fibrillarin | *123 (85-154)* | *77 (51-97)* |
