# Supplementary material for: Systemic Sclerosis in Zimbabwe: Autoantibody Biomarkers, Clinical, and Laboratory Correlates
Source: Front Immunol. 2021 Nov 9;12:679531. doi: 10.3389/fimmu.2021.679531 (PMC8631108; doi:10.3389/fimmu.2021.679531)
Supplement: Supplementary Table 2 — Comparison total Immunoglobulin IgA, IgG, IgM concentrations in patients with different SSc specific autoantibodies. [file Table_2.docx]

**Supplementary Table 2:**

**Comparison total Immunoglobulin IgA, IgG, IgM concentrations in patients with different SSc specific autoantibodies.**

|  | *IgA mg/dL* | *IgM mg/dL* | *IgG mg/dL* | *IgE (kU/L)* |
| --- | --- | --- | --- | --- |
| **All patients** | ***264 (21.9-698.9)*** | ***122.67 (0.5 -680)*** | ***1315 (104-2780)*** | ***187.8 (7-615)*** |
| **Scl-70** | ***320.34 (167-501)*** | *130.8 (78-205)* | ***1717 (1279-2430)*** | *51.3 (41-311)* |
| **PMScl 100** | *293.44 (159-533)* | *128 (28-444)* | ***1492 (1089-2780)*** | *213 (36-516)* |
| **PM-Scl75** | *253.65(133-464.)* | *95.73 (28-295)* | *1294 (971-1857)* | *188.9 (7-500)* |
| **Ku** | *294.35 (191-473)* | *126 (44-282)* | *1217 (877-1623)* | *166.8 (7-500)* |
| **Fibrillarin** | ***335.6 (165-679)*** | *97 (31-231)* | *1221 (904-1448)* | *175.5 (22-500)* |
| **NOR90** | *260 (159-366)* | *80.6 (28-206)* | *1276 (866-1858)* | *116 (7.4-225)* |
| **Th/To** | *265.9 (63-688)* | *98.9 (28-231)* | *1306 (930-2185)* | *174 (22-165)* |
| **RNAP11** | *265.5 (153-415)* | *141 (11-444)* | *1403 (880-2724)* | *210 (7.4-340)* |
| **RNAP155** | *286.7 (24-699)* | *261.8 (0.5-98)* | *1347(866-2725)* | *175.5 (7-490)* |
| **CENP-B** | *294.5 (130-523)* | *105.6 (23-154)* | *1310 (939-2780)* | *177 (8.3-500)* |
| **Ro-52** | ***325.2 (185-533)*** | *116.9 (31-312)* | *1385 (11078-1799)* | *111.4 (7-340)* |
