# Supplementary material for: Systemic Sclerosis in Zimbabwe: Autoantibody Biomarkers, Clinical, and Laboratory Correlates
Source: Front Immunol. 2021 Nov 9;12:679531. doi: 10.3389/fimmu.2021.679531 (PMC8631108; doi:10.3389/fimmu.2021.679531)
Supplement: Supplementary file 3 [file Table_3.docx]

| Vasculopathy | Raynaud's Vasculopathy | 57 | 24% |
| --- | --- | --- | --- |
| Cardiovascular |  |  |  |
|  | Chest pain | 26 | 11% |
|  | Retrosternal pain | 15 | 6.25% |
|  | Palpitations | 17 | 7% |
|  | Congestive cardiac failure (n=2), | 6 | 2.5% |
|  | Other:(murmur=3, infarction =2, effusion=3valve disease=3) | 12 | 4.85% |
|  | Hypertension | 10 | 4% |
| Systemic symptoms |  |  |  |
|  | lymphadenopathy | 16 | 6.5% |
|  | tiredness | 17 | 7% |
|  | sweating | 7 | 3% |
|  | exhaustion | 7 | 3% |
|  | feeling drained | 5 | 2% |
|  | fever or chills | 7 | 3% |
| Ocular |  |  |  |
|  | Itchiness of the eyes | 84 | 35% |
|  | Redness of the eyes | 43 | 18% |
|  | Sand grain sensation | 37 | 12.5% |
|  | Allergic conjunctivitis | 18 | 7.5% |
|  | Gritty eye sensation | 16 | 6.7% |
|  | Discharge | 15 | 6.3% |
|  | Allergic conjunctivitis | 18 | 7.5% |
|  | Gritty eye sensation | 16 | 6.7% |
|  | Discharge | 15 | 6.3% |
|  | Swollen, baggy, puffy eyes | 13 | 5.4% |
| Neurological |  |  |  |
|  | Headaches, including migraine | 74 | 31% |
|  | carpal tunnel syndrome | 7 | 3% |
|  | forgetfulness | 6 | 2.5% |
|  | anosmia | 5 | 2% |
|  | numbness | 5 | 2% |
|  | Other neurological (paraesthesia, sensory loss, forgetfulness, confusion, memory loss) | 19 | 8% |
| Psychiatric |  |  |  |
|  | psychosis | 3 | 1.25% |
|  | anxiety | 3 | 1.25% |
|  | Attention deficit disorder | 1 | 0.4% |
|  | nightmares | 1 | 0.4% |
| Endocrine |  |  |  |
|  | Diabetes mellitus (n=8) | 8 | 3% |
|  | insulin resistant diabetes mellitus (n=3), | 3 | 1.25% |
|  | hypothyroidism (n=7), | 7 | 3% |
|  | hyperthyroidism (n=7), | 7 | 3% |
|  | parathyroid adenoma (n=3) | 3 | 1.5% |
| Gynecological | |  |  |
|  | menstrual irregularities (n=6), | 6 | 2.5% |
|  | fibroids (n=2), | 2 | 0.8% |
|  | History of hysterectomy (n=2), | 2 | 0.8% |
|  | premature menopause at 28 years (n=1), polycystic ovary syndrome (n=1) | 2 | 0.8% |
| Malignancy |  |  |  |
|  | Cancer of the uterine cervix (2), | 2 | 0.8% |
|  | Breast cancer (2), | 2 | 0.8% |
|  | Kaposi sarcoma (1), | 1 | 0.4% |
|  | Non-Hodgkin's lymphoma (1) | 1 | 0.4% |
| Renal |  |  |  |
|  | Proteinuria | 4 | 1.6% |
|  | Leukocyturia | 3 | 1.5% |
|  | Hematuria | 3 | 1.5% |
|  | Kidney Cysts | 2 | 0.8% |
|  | Malignant Hypertension (N=1) and Bilirubinuria (N=1) | 2 | 0.8% |
| Hematological | |  |  |
|  | anemia (n=2) | 2 | 0.8% |
|  | von Willebrand's disease, (n=1), | 1 | 0.4% |
| Immunology/Autoimmune | |  |  |
|  | Sjogren’s Disease (n=1) | 1 | 0.4% |
|  | Myeloperoxidase (MPO) antibody positivity (n=1) | 1 | 0.4% |
|  | myasthenia gravis (n=1) | 1 | 0.4% |
| Infectious diseases | |  |  |
|  | Schistosoma haematobium | 2 | 0.8% |
|  | Candida albicans (1), Gram positive staphylococcus (1) | 2 | 0.8% |
| Occupational (Patch Test positivity) |  |  |  |
|  | Epoxy Resin (n=1), Thiuram mix (n=1), Potassium dichromate (n=1) Methyl Paraben (n=1), | 4 | 1.6% |

**Supplementary Table 3**

Frequency and types of cardiovascular, renal, ocular neurological and other symptoms diagnosed in patients with SSc specific autoantibodies.
